# New Sample Preparation Method for Honey Volatiles Fingerprinting Based on Dehydration Homogeneous Liquid–Liquid Extraction (DHLLE)

**DOI:** 10.3390/molecules23071769

**Published:** 2018-07-19

**Authors:** Piotr M. Kuś, Igor Jerković

**Affiliations:** 1 Department of Pharmacognosy, Wrocław Medical University, ul. Borowska 211a, 50-556 Wrocław, Poland; 2 Department of Organic Chemistry, Faculty of Chemistry and Technology, University of Split, Ruđera Boškovića 35, 21000 Split, Croatia; igor@ktf-split.hr

**Keywords:** green sample preparation method, honey volatiles, terpenes, benzene derivatives

## Abstract

Qualitative chemical fingerprinting of the honey volatiles by gas chromatography and mass spectrometry (GC-MS) has been an efficient authentication tool that allowed for the classification of the honey botanical origin (strongly related to its medicinal and market value). However, the usage of current sample preparation methods is limited by selectivity of the volatiles extraction from the honey matrix and requires significant solvent volume. Therefore, a new sample preparation method based on dehydrating homogeneous liquid–liquid extraction (DHLLE) involving reduced solvent usage was developed for screening volatiles and semi-volatiles from the honey. The effective extraction was achieved by implementing a miscible liquid extraction system (aqueous honey solution/isopropanol) followed by separation through dehydration with MgSO_4_ and purification by a solvent polarity change and washing. The method was evaluated by estimating accuracy and precision. The DHLLE method showed satisfactory recoveries (75.2 to 93.5%) for typical honey volatiles: linalool, borneol, terpinen-4-ol, α-terpineol, *p*-anisaldehyde, eugenol, and vanillin. It also showed superior repeatability with percent relative standard deviation (RSD%) 0.8–8.9%. For benzyl alcohol, methyl syringate, and caffeine, the recoveries were 54.3 to 63.9% and 67.3 to 77.7% at lower and higher spiking levels, respectively. Applied to unifloral apple honey, the DHLLE method allowed for the identification of 40 compounds including terpenes, hydrocarbons, phenylpropanoids, and other benzene derivatives, which makes it suitable for fingerprinting and chemical marker screening. The obtained results were comparable or better than those obtained with ultrasonic extraction with dichloromethane.

## 1. Introduction

Honey is an important medicinal substance and food product with production of around 250,000 tons per year in the European Union (EU), the second world producer [1]. Besides nutritional value, honey exhibits various biological properties valuable in medical applications i.a. wound-healing, antibacterial activity (even against antibiotic-resistant strains) [2,3], or antioxidant activity [4,5]. Since the major concern is correct classification of the honey botanical origin (strongly related to its medicinal and market value), the EU has been encouraging its proper geographical and botanical origin labeling as well as the authentication methods development [6,7]. Moreover, the EU has supported the development of green methods in different fields as part of improving sustainable development and pollution/waste minimization.

Honey is a complex mixture consisting of small amounts of numerous compound classes distributed in a viscous matrix of supersaturated carbohydrate solution. Aroma-active compounds have been particularly interesting for authentication since they are strictly related to the honey botanical origin as well as to the sensory properties [8,9]. However, due to their low amounts and large variety of different compound classes as well as high honey viscosity, the extraction of volatile organic compounds (VOCs) from honey remains a challenge. To date, various methodologies have been developed and used for VOCs isolation such as liquid–liquid extraction (LLE), solid phase extraction (SPE), solid phase micro-extraction (SPME), dynamic headspace (DHS), headspace solid-phase micro-extraction (HS–SPME), ultrasonic solvent extraction (USE), hydrodistillation (HD), and micro simultaneous steam distillation–solvent extraction (MSDE). HS–SPME, USE, HD, and MSDE have been evaluated by Alissandrakis et al. [10]. Both HD and MSDE, which are very common techniques for VOCs isolation from other matrices, were not suitable for the honey since the drastic conditions used, led to the degradation of some compounds and the formation of artifacts. The most useful were found to be USE, which is an improved LLE technique. This method provided the most representative VOCs profile since no heat was applied. In addition, HS–SPME was found to be suitable as well. However, it turned out to be very selective and the obtained headspace profile was greatly affected by the fiber type. Furthermore, it was not appropriate for semi-volatiles [10]. Castro-Vázquez et al. compared MSDE, LLE, and SPE methods concluding that LLE is the recommended method since it provided good yield as well as low standard deviations for most of the compounds analyzed [11]. Performance of SPE was generally similar to LLE except for lower recovery of esters and higher standard deviations. More recently, Castro-Vázquez et al. proposed an optimized extraction method for honey VOCs by SPE, which allowed the effective removal of interfering substances and VOCs extraction provided high recoveries of the standard compounds that ranged 56.5 to 102.1% with relative standard deviation RSD% lower than 6% [12].

In recent years, more attention has been paid to the environmental imprint from the human presence. An urgent need for sustainability is raised. Therefore, in the last few years, intensified efforts were made not only to develop time-efficient and cost-efficient methodologies but also to develop greener extraction and sample preparation methods [13]. From this point of view, HS–SPME is efficient but also very selective and, therefore, it does not provide complete and representative results. Often, it has been accompanied by other techniques to obtain comprehensive data. The best methods providing the most representative profiles of the honey volatile and semi-volatile compounds are USE and SPE. However, those techniques are not only time consuming but also require a high amount of solvents (ca. 60 mL of dichloromethane or pentane/diethyl ether per sample of 20–40 g) [12,14,15] as well as energy used for heating during the extracts concentration. Dichloromethane is considered problematic or hazardous and pentane and diethyl ether are considered highly hazardous solvents [16]. Moreover, single-use SPE cartridges are not only expensive but their utilization generates additional waste along with used solvents. From an ecological point of view, the cost of both methods including volume of solvents used for the extraction, generated waste, and energy consumption is relatively high and reduces their availability for routine analyses. 

Since LLE methods provide the ability to extract a wide spectrum of compounds, development of small solvent volume LLE techniques is preferred. Anastassiades et al. proposed a two-step method of pesticide residues extraction based on QuEChERS (which stands for quick, easy, cheap, effective, rugged, and safe) principles [17]. The method is based on extraction/partitioning with a water-soluble solvent (acetonitrile) and dehydration with MgSO_4_ as well as subsequent clean-up by dispersive SPE using a primary secondary amine sorbent (PSA). More recently, Angioni et al. proposed a simple, ecological, and cost-efficient method for the volatile wine aroma compound isolation by dehydration and separation of the alcoholic-glycerine layer based on the principle of homogenous liquid–liquid extraction and use of the natural composition of the wine [18]. To the best of our knowledge, a similar approach hasn’t been applied to extract the honey volatiles. The honey matrix provides additional difficulties related to predominant carbohydrate content. Therefore, the focus of the present study was to develop a cost-efficient method based on small volume homogenous liquid–liquid extraction adapted for the extraction of volatile and semi-volatile compounds from honey that could be used for effective, qualitative screening. This includes selective extraction and purification of the extracts to avoid an excess of very polar and very hydrophobic interfering compounds (which result as baseline shifts, covering peaks, and poor MS spectra quality in gas chromatography and mass spectrometry (GC-MS)) irrelevant for the honey characterization. To date, the analyses of honey VOCs have been focused on the qualitative chemical fingerprinting and identification of characteristic compounds including specific and non-specific chemical biomarkers. Therefore, the priority for the new method development was to reduce the amount of the sample and solvents used as well as to manage reasonable repeatability and recovery. To test the developed method, an artificial honey containing solution of sugars and selected volatile compounds of different polarity and structure was used, similarly to previous studies [12]. The compounds were selected to cover the spectrum of different chemical classes relevant for honey authentication (mainly terpenes and benzene derivatives but also phenylpropanoids and caffeine) [9,15,19,20,21]. The recoveries of artefacts (HMF) or higher aliphatic hydrocarbons were not investigated since they are irrelevant for honey authentication [22,23]. The method was also applied to a real apple blossom honey sample, which is naturally rich in a large variety of VOCs belonging to the different chemical classes of natural organic compounds frequently occurring in various honey types.

## 2. Materials and Methods

### 2.1. Materials and Samples

Isopropanol, dichloromethane, anhydrous MgSO_4_, and Na_2_SO_4_, which all had analytical purity, were supplied by Chempur (Piekary Śląskie, Poland). Terpinen-4-ol, α-terpineol, linalool, borneol, eugenol, cedrol, caffeine, *p*-anisaldehyde, and vanillin were obtained from Sigma-Aldrich (Darmstadt, Germany). Benzyl alcohol was obtained from Merck (Darmstadt, Germany). Methyl syringate was prepared in the laboratory according to Hristea [24], purified by multiple re-crystallization, and evaluated by liquid chromatography-diode array detector- mass spectrometry (LC-DAD-MS). The reference compounds were all ≥98–99% purity.

The apple blossom (*Malus domestica* Borkh.) honey sample from Poland was obtained from a professional beekeeper and classified as unifloral based on the beekeeper’s declaration as well as by the pollen-analysis (>45% *Malus* spp. pollen). The latter was performed by examining in a Hundh 500 (Helmut Hund GmbH, Wetzlar, Germany) light microscope coupled to a digital camera (Motic m 1000, Motic, Hong Kong, China) and an image analysis system (Motic images plus software) for morphometry of the pollen grains. The sample was stored in a hermetically closed glass bottle at 4 °C until the VOCs isolation. Artificial honey (a honey-like sugar matrix) containing 448 g/L fructose and 350 g/L glucose was prepared according to the research from Castro-Vázquez et al. [12] and spiked with reference standards (Table 1) representing different groups of compounds naturally occurring in various types of honey at two levels of concentration: 300 µg/L for α-pinene, terpinen-4-ol, α-terpineol, linalool, borneol, 600 µg/L for *p*-anisaldehyde, eugenol, vanillin, methyl syringate, caffeine, and 1000 μg/L for benzyl alcohol as well as at two-fold higher concentration for all of these compounds. In addition, 3 µL of cedrol solution (1 mg/mL) was used as the internal standard. The stock standard solutions (1 mg/mL) of the reference compounds were prepared in methanol and stored in the dark at −20 °C. The spiking solutions were prepared prior to the analysis by diluting it with methanol to achieve an appropriate concentration. A volume of 20 μL of standard solution was mixed with the sugar matrix.

### 2.2. Dehydration Homogenous Liquid–Liquid Extraction Method

A total of 5 g of the honey (or artificial honey spiked with reference compounds) was weighed in a 15 mL centrifuge tube and carefully diluted with 6 mL of ultrapure water using a vortex mixer (Lab dancer, IKA, Königswinter, Germany). In addition, 2 mL of isopropanol was added subsequently and the solution was mixed. Afterwards, 6 g of MgSO_4_ was added in small portions to dehydrate the sample. The tube was cooled in a cold water bath. The content of the tube was subsequently mixed for 30 s using a stainless steel spatula to achieve a homogeneous suspension. The tube was centrifuged for 5 min at 3000 rpm to separate two phases. The upper isopropanol phase was transferred to another tube. To clean the extract from the rest of the carbohydrates and traces of other polar contaminants, the alcoholic phase was diluted with 1 mL of dichloromethane and washed twice with 1 mL of ultrapure water. The remaining extract in the dichloromethane-isopropanol solution was additionally dried by filtering through anhydrous Na_2_SO_4_. The extract was carefully concentrated to a volume of 200 µL by evaporating the excessive solvent using a fractionating column. Furthermore, 2 µL were injected to the gas chromatography-flame ionization detector/mass spectrometry (GC-FID/MS) system.

### 2.3. Dehydration Homogenous Liquid–Liquid Extraction Method Evaluation

To evaluate the DHLLE method, its accuracy and precision was assessed. To determine the accuracy, the spike recovery method was applied. The reference compounds with known amounts include terpinen-4-ol, α-terpineol, linalool, borneol, eugenol, caffeine, *p*-anisaldehyde, vanillin, benzyl alcohol, and methyl syringate (concentrations mentioned above), which were recovered from the artificial honey matrix using the dehydrating homogeneous liquid–liquid extraction (DHLLE) method. The whole extraction procedure of spiked artificial honey at two levels of concentration was repeated 5 times as well as the DHLLE method for the real, not-spiked honey sample. The internal standard of 3 µL of cedrol solution (1 mg/mL) was added. The accuracy was calculated and expressed as a percent recovery. To evaluate precision, repeatability was calculated and expressed as the percentage relative standard deviation (RSD%) of the GC chromatogram peak area values obtained for spiked artificial honey (Table 1). Similarly, apple honey extracts were analyzed and the RSD percentage was calculated for the compounds identified in the real honey sample. 

### 2.4. Ultrasonic Solvent Extraction 

To compare the results obtained by the DHLLE with another ubiquitous and comparable method, volatile and semi-volatile compounds were also extracted by USE using an ultrasound bath (Elmasonic Typ S 30 H, Elma Schmidbauer GmbH, Singen, Germany) at 25 ± 3 °C. Forty grams of the honey sample were dissolved in 22 mL of ultrapure water and 1.5 g of anhydrous Na_2_SO_4_ was added. Dichloromethane was used for the extraction. The honey solution was extracted in triplicate with fresh portions of the solvent (20 mL) for 30 min in the ultrasound bath. The organic layers were filtered over anhydrous Na_2_SO_4_ and joined and concentrated to 200 µL by distillation with a Kuderna-Danish concentrator. The applied procedure was similar to previously published research [15,25,26]. For GC-FID and GC-MS analyses, 2 μL of the extract was used.

### 2.5. Chromatographic Conditions 

The GC-FID analyses were performed with an Agilent Technologies (Palo Alto, CA, USA) gas chromatograph model 7890A equipped with an FID detector and an HP-5MS capillary column (5% phenyl-methylpolysiloxane, 30 m, 0.25 mm i.d., coating thickness 0.25 μm, Agilent). The GC conditions were similar to those described previously [26]. The oven temperature was programmed isothermal at 70 °C for 2 min, increased from 70–200 °C at 3 °C·min^−1^, and held isothermally at 200 °C for 15 min for a carrier gas known as He (1.0 mL·min^−1^). The injector temperature was 250 °C and the FID detector temperature was 300 °C. The GC-MS analyses were carried out using an Agilent Technologies gas chromatograph model 7820A equipped with a mass selective detector (MSD) model 5977E (Agilent Technologies) and an HP-5MS capillary column under the same conditions as for the GC-FID analysis. The MSD (EI mode) was operated at 70 eV, the ion source temperature was 230 °C, and the mass range was 30–300 amu, which was previously reported [26]. The identification of the VOCs was based on the comparison of their retention indices (RI) determined relative to the retention times of *n*-alkanes (C_9_-C_25_) with those reported in the literature [15,26,27,28] and their mass spectra with authentic compounds were available in our laboratories or those listed in Wiley 9 (Wiley, New York, NY, USA) and NIST 14 (Gaithersburg, MD, USA) mass spectral libraries. The VOCs percentage composition was computed from the GC peak areas using the normalization method (without correction factors).

## 3. Results and Discussion

Since honey contains a wide spectrum of volatile and semi-volatile compounds with various polarities, a homogenous liquid–liquid extraction-based approach was developed for their extraction with the use of water-miscible solvents involving dissolution in water, dehydration with MgSO_4_, centrifugation, and a cleaning step. A honey matrix contains high carbohydrate content. Therefore, selection of the extraction solvent should consider low sugar extraction and water-sugar system miscibility. After the preliminary study and experiments with various solvents (data not shown), isopropanol (IPA) was selected as a suitable water-miscible and environmentally-friendly extraction solvent providing enough high selectivity towards the VOCs. Isopropanol exhibited high affinity for the most relevant VOCs (mostly oxygenated compounds). The selected extraction solvent is recommended and characterized as a green solvent and, therefore, may successfully substitute some of the more toxic solvents [29,30]. Isopropanol is one of the less polar liquids among the most common fully water-miscible solvents (at room temperature) with a Snyder polarity index of 4.3 (for water and dichloromethane it is 9.0 and 3.4, respectively) [31,32]. The miscibility of IPA with water allowed highly efficient and fast extraction by surpassing the limitations of the contact surface area present in non-miscible solvent systems of classical LLE. Additionally, binding of the water by MgSO_4_ not only allows the separation of the solvents, but it also reduces the solubility of the compounds of interest in the water layer. To provide efficient extraction and separation of the organic layer, the most suitable proportions found were 5 g of honey, 6 mL of water, 2 mL of isopropanol (IPA), and 6 mg of MgSO_4_. Extra low amounts of water and a high quantity of MgSO_4_ increased the density of the obtained mixture, which impeded the solvent separation. However, a part of non-volatile polar compounds likely remained in the isopropanol extract, which resulted in a relatively poor quality of chromatogram. Therefore, the purification step was introduced.

One of the study objectives was to focus on the most representative groups of VOCs needed for honey fingerprinting and preferably cleaning them from an excess of unwanted molecules. They include more polar compounds (traces of sugars and their derivatives) as well as an excess of higher aliphatic compounds (derived from the comb [23]) that interfere with the analyses and do not contribute to the characterization of the honey type, which may be observed as baseline shifts, enormous peaks, and a drop of overall chromatogram quality. Therefore, the methodology including the cleaning step and solvent polarity modifications were adopted. As a purification step, the addition of a very small quantity of dichloromethane was necessary (no appropriate and efficient “green” substitute was found) to slightly change the polarity of isopropanol extract without forming a separate phase. This allowed to wash the extracts with water since the obtained mixture formed the LLE system consisting of separate water and organic solvent layers. Subsequent partitioning of the layers allowed for the successful removal of unwanted, polar compounds. As the result, the amount of interfering groups of compounds has been reduced, while most of the interesting compounds were retained, allowing to obtain good quality chromatogram. Moreover, a part of isopropanol was removed from the dichloromethane-isopropanol system by washing with water, which allowed for a simpler extract concentration. The application of IPA as an extraction solvent allowed for an efficient extraction of a wide range of compounds (Table 2). In comparison to the direct extraction of VOCs with a typically used solvent (dichloromethane), several more compounds were found.

### 3.1. Performance of the Extraction Method

Table 1 presents the obtained results of the precision and accuracy of the DHLLE method for selected representative VOCs occurring naturally in various honey types described in the literature. The proposed method provides superior recovery (ranging from 75.2 to 93.5%) for most of the selected compounds at a higher level of concentrations except for caffeine (67.3%). At a lower level of concentration, high yields (76.9–91.7%) were obtained for most compounds except for methyl syringate (54.3%), caffeine (57.0%), and benzyl alcohol (63.9%). The results were also quite repeatable with RSD% from 0.8 to 8.9% at a lower spiking concentration level and from 1.7 to 5.6% at a higher concentration level. Therefore, for compounds such as linalool, borneol, terpinen-4-ol, α-terpineol, *p*-anisaldehyde, eugenol, and vanillin, it may meet SANTE/11813/2017 requirements [33]. The levels of recovery are also satisfactory for fingerprinting and qualitative screening. As expected, the highest recoveries were obtained for more hydrophobic compounds such as terpenes, *p*-anisaldehyde, and eugenol and were lower for the more polar constituents e.g., caffeine, 2-phenylethanol due to less efficient extraction and/or partial loss during the clean-up step that included washing with water.

**Figure 1:**
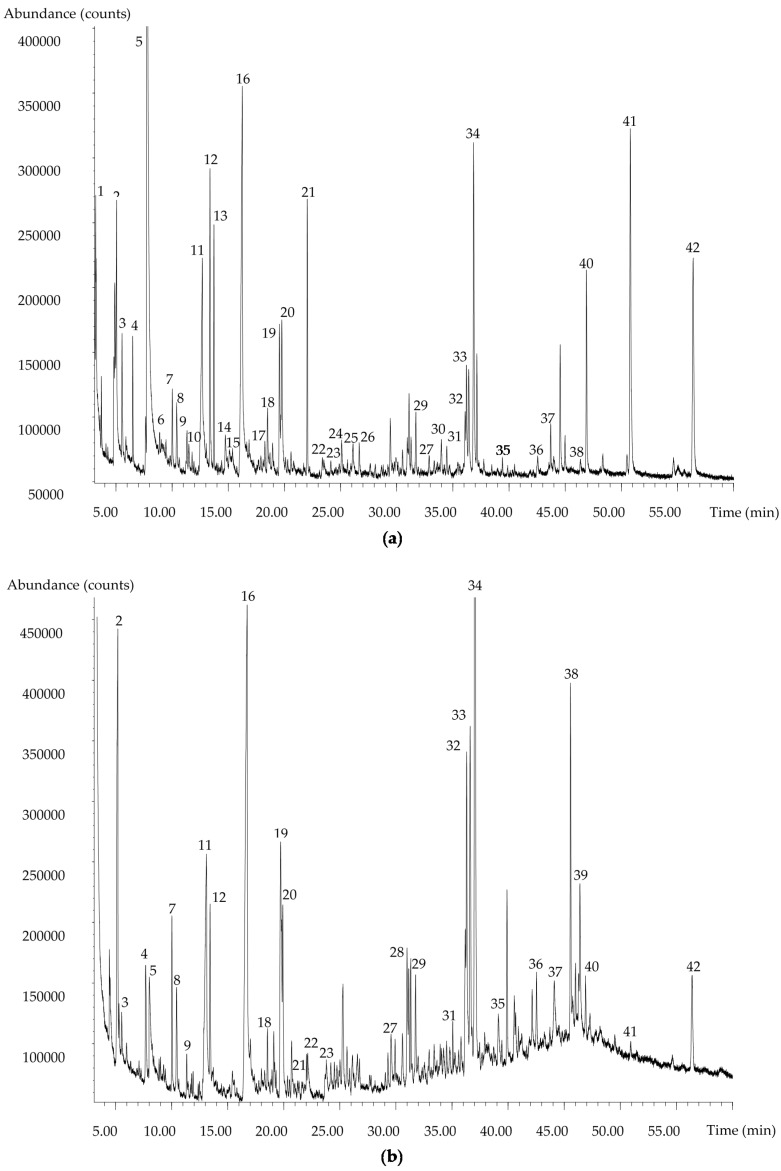
Comparison of representative total ion chromatograms of apple honey volatiles extracts obtained using the proposed DHLLE method (**a**) and the USE extraction with CH_2_Cl_2_ (**b**). The numbers correspond to the compounds listed in Table 2.

To further investigate the suitability of the method, it was applied to a real apple blossom honey sample containing a large variety of VOCs chemical classes [34]. The DHLLE method allowed for the extraction and identification of 40 compounds including terpenes, norisoprenoids, phenylpropanoids, and other benzene derivatives, indole derivatives, furan derivatives, and aliphatic compounds (Table 2, Figure 1). The results obtained for the honey sample demonstrate a relatively high repeatability for most of the compounds such as terpenoids or benzene derivatives with RSD% ranging from 1.7 (terpendiol I) to 11.0% (vanillin). The values are just slightly higher than in spiked artificial honey solution. The main identified components in terms of area percentage were vinyl caproate (22.4%) and phenylacetic acid (13.8%). In comparison with the dichloromethane extract (Table 2, Figure 1), the proposed method allowed the identification of nine more compounds. The difference is noted mainly for more polar compounds such as acids and furan derivatives, e.g., 3-methylbutanoic acid or vanillic acid as well as 2,3-dihydro-3,5-dihydroxy-6-methyl-4*H*-pyran-4-one, 2,3-dihydrobenzofuran, HMF, 1*H*-indole, dodecan-1-ol, *p*-hydroxybenzoic acid, and phenylacetaldehyde. The percentage of vinyl caproate in the DHLLE extract was much higher than in the USE extract. However, aliphatic compounds as well as vomifoliol were present in smaller percentages in the DHLLE extracts than in the USE extracts and two of them (hexadecane, octadecan-1-ol) were identified only in the latter. The chromatogram of the DHLLE extract (Figure 1) appears more equilibrated in all the retention time ranges and provides an almost flat baseline, which confirms high sample purity. The quality of the chromatogram is satisfactory for the identification of the relevant compounds present in the extract. In comparison, the chromatogram of the USE extract demonstrates marked baseline drifts. Considering a much lower quantity of the sample and solvent used (2 mL IPA + 1 mL CH_2_Cl_2_ vs. 60 mL CH_2_Cl_2_), the results obtained by the DHLLE method are more efficient. Additionally, since all the relevant compounds as well as some additional ones were extracted, this method could be considered an improved substitute of the USE extraction suitable for honey volatiles fingerprinting.

### 3.2. Comparison with Literature Data on Ultrasonic Solvent Extraction and Solid-Phase Extraction Methods

Compared to other methods such as USE or SPE, it can be observed that similar compound classes including benzene derivatives, oxygenated terpenes, norisoprenoids, and aliphatic compounds such as hydrocarbons, aliphatic acids, and alcohols, furan derivatives, etc. are extracted by all of them [12,14]. The recovery of selected compounds were not evaluated for the USE and the SPE extraction of the volatiles was comparable or higher (56.5–102.1%) [12]. The repeatability reported in the present study (1.7–8.9% for spiked samples) in terms of the RSD percentage was similar to the results reported for the SPE method by Castro-Vázquez et al. (0.6–5.6%) [12]. The RSD percentage values reported for the USE by Alissandrakis et al. were relatively higher (7.2–23.6%) [14], which was similar to the obtained results for USE (1.1–26.6%).

Comparing the three methods in terms of the sample and reagents use, the DHLLE method provides a significant advantage. The amount of honey (40 g, USE; 20 g, SPE; 5 g, DHLLE) and the volume of organic solvents (60 mL CH_2_Cl_2_, USE; 25 mL MeOH and 60 mL CH_2_Cl_2_, SPE; 2 mL isopropanol, and 1 mL of CH_2_Cl_2_, DHLLE) can be significantly reduced by applying the new method. Additionally, in the DHLLE method, the main solvent used is a less toxic isopropanol. Instead of 60 mL of CH_2_Cl_2_ (volume of solvents needed for SPE column conditioning not included) in the SPE or USE, it may be reduced to 3 mL (2 mL isopropanol and 1 mL of dichloromethane) proposed in the current research. Additionally, the use of expensive sorbents as well as distillation of a large amount of solvents are avoided. This provides not only a reduction of the analysis cost and environmental imprint, but also of the sample preparation time and makes the procedure available and interesting for routine analyses.

## 4. Conclusions

A new proposed DHLLE method allows fast, repeatable, and cost-efficient extraction of the honey volatiles covering a broad spectrum of volatile and semi-volatile compounds of different polarities. The amount of sample and solvents used was low but keeping reasonable repeatability and recovery for the standards of volatiles with different polarities based on testing in artificial honey. 

The DHLLE method provides good quality GC chromatograms and may be applied for routine analyses of the honey phytochemical profiles (sample screening, qualitative chemical fingerprinting, and detection of major chemical markers) that was confirmed by the application of the DHLLE method to apple honey. Additionally, the method allows significant minimization of the reagents used in comparison with other methods such as USE or SPE, which makes it more cost and time-efficient as well as environmentally-friendly (greener) based on QuEChERS principles.

## Figures and Tables

**Table 1:** The percentage of recovery and percent relative standard deviation (RSD%) of the volatile compounds extracted from a sugar model solution (artificial honey) using the dehydrating homogeneous liquid–liquid extraction (DHLLE) method.

Compound	RI ^1^	Concentration Level A (*n* = 5)			Concentration Level B (*n* = 5)		
		Recovery ^2^ (%)	SD	RSD%	Recovery ^2^ (%)	SD	RSD%
Benzyl alcohol	1039	63.9	3.0	4.8	77.7	2.6	3.4
Linalool	1100	83.7	3.4	4.0	89.0	4.9	5.5
Borneol	1174	76.9	3.8	5.0	82.0	4.1	5.0
Terpinen-4-ol	1180	76.9	3.5	4.6	79.2	2.9	3.7
α-Terpineol	1193	85.7	4.7	5.5	85.9	2.1	2.4
*p*-Anisaldehyde	1256	91.7	4.8	5.3	93.5	5.3	5.6
Eugenol	1361	78.7	4.3	5.5	75.2	3.2	4.3
Vanillin	1403	87.6	0.7	0.8	77.9	3.8	4.9
Methyl syringate	1773	54.3	3.8	7.1	75.2	1.3	1.7
Caffeine	1843	57.0	5.1	8.9	67.3	3.7	5.5

Concentration level: terpinen-4-ol, α-terpineol, linalool, borneol (A: 300 µg/L; B: 600 µg/L), *p*-anisaldehyde, eugenol, vanillin, methyl syringate, caffeine (A: 600 µg/L; B: 1200 µg/L), benzyl alcohol A: 1000 µg/L; B: 2000 µg/L). ^1^ RI: Retention indices determined relative to *n*-alkanes (C_9_–C_25_) on HP-5MS column (Agilent Technologies, Palo Alto, CA, USA), ^2^ average percentage (*n* = 5), SD: standard deviation.

**Table 2:** The comparison of volatile profiles obtained by the DHLLE and ultrasonic solvent extraction (USE) with dichloromethane.

No.	Compound	RI ^1^	DHLLE			USE		
			Av. (Area %) ^2^	±SD	±RSD%	Av. (Area %) ^2^	±SD	±RSD%
1	3-Methylbutanoic acid (Isovaleric acid)	<900	7.9	0.8	10.4	nd	-	-
2	3-Methylpentanoic acid	942	3.9	0.2	5.2	8.1	0.5	6.3
3	Benzaldehyde	966	1.2	0.0	3.8	0.8	0.1	9.9
4	Benzyl alcohol	1039	0.6	0.0	4.9	2.1	0.1	4.3
5	Vinyl caproate ^3^	1048	22.4	1.6	7.2	4.0	0.2	4.2
6	Phenylacetaldehyde	1049	0.5	0.0	6.0	nd	-	-
7	Hotrienol	1106	0.8	0.1	8.9	1.9	0.1	3.1
8	2-Phenylethanol	1115	0.9	0.1	5.6	1.8	0.2	8.9
9	2-Phenylacetonitrile	1140	0.4	0.0	6.5	0.7	0.1	20.2
10	2,3-Dihydro-3,5-dihydroxy-6-methyl-4*H*-pyran-4-one	1142	0.5	0.0	5.0	nd	-	-
11	Benzoic acid	1162	6.8	0.2	3.4	8.6	0.4	4.8
12	3,7-Dimethylocta-1,5-diene-3,7-diol (Terpendiol I)	1191	3.4	0.1	1.7	2.3	0.3	13.9
13	Dodecane	1200	1.8	0.1	1.1	nd	-	-
14	2,3-Dihydrobenzofuran	1222	0.5	0.1	10.6	nd	-	-
15	HMF	1230	0.5	0.0	6.3	nd	-	-
16	Phenylacetic acid	1251	13.8	1.3	9.2	18.2	0.5	2.7
17	1*H*-Indole	1295	0.5	0.0	7.0	nd	-	-
18	4-Vinyl-2-methoxyphenol	1314	0.6	0.1	11.0	0.8	0.0	3.7
19	3-Hydroxy-4-phenylbutan-2-one	1352	2.1	0.1	2.4	1.0	0.1	8.9
20	(*E*)-8-Hydroxylinalool	1367	0.4	0.0	6.1	1.0	0.1	11.4
21	Tetradecane	1400	3.1	0.1	3.7	0.3	0.1	25.8
22	4-Hydroxy-3-methoxybenzaldehyde (Vanillin)	1403	0.6	0.1	11.0	1.3	0.3	24.4
23	*trans*-Cinnamic acid	1434	1.9	0.1	6.9	1.5	0.4	26.6
24	Dodecan-1-ol	1479	0.8	0.1	5.9	nd	-	-
25	*p*-Hydroxybenzoic acid	1517	0.2	0.0	7.7	nd	-	-
26	4-Hydroxy-3-methoxybenzoic acid (Vanillic acid)	1567	0.4	0.0	1.4	nd	-	-
27	5-Amino indanone	1594	1.1	0.1	4.6	1.1	0.0	2.6
28	Hexadecane	1600	nd	-	-	0.8	0.1	7.1
29	3-Hydroxy-β-damascone	1617	0.7	0.0	5.2	1.1	0.2	19.2
30	3-Hydroxy-4-phenyl-2(5*H*)-furanone	1695	0.7	0.1	9.0	nd	-	-
31	6,7-Dehydro-7,8-dihydro-3-oxo-α-ionol	1733	0.9	0.0	2.4	0.7	0.1	18.8
32	9-Hydroxymegastigma-4,6-dien-3-one	1769	1.0	0.1	5.8	2.1	0.4	21.7
33	Methyl syringate	1773	2.7	0.1	5.2	5.4	1.2	22.5
34	4-Hydroxy-3,5,5-trimethyl-4-(3-oxo-1-butenyl)-2-cyclohexen-1-one (Vomifoliol)	1803	4.8	0.2	3.7	17.5	0.7	4.0
35	Hexadecan-1-ol	1882	0.4	0.0	10.9	2.4	0.1	3.9
36	Hexadecanoic acid	1966	1.0	0.1	4.7	1.1	0.0	2.7
37	Methyl indole-3-acetate	1980	1.1	0.1	7.2	2.0	0.5	23.3
38	(*Z*)-Octadec-9-en-1-ol	2059	0.5	0.0	7.9	4.9	0.1	1.1
39	Octadecan-1-ol	2084	nd	-	-	1.0	0.0	4.2
40	Heneicosane	2100	1.2	0.0	2.7	0.8	0.0	4.7
41	Octadecanoic acid	2181	4.0	0.2	5.9	0.6	0.1	21.5
42	Tricosane	2300	2.6	0.3	9.5	1.6	0.1	4.3

^1^ RI: Retention indices determined relative to *n*-alkanes (C_9_-C_25_) on the HP-5MS column, ^2^ average area percentage (*n* = 5), ^3^ tentatively identified, nd: not detected.
